# Supplementary material for: Multi-layered heterochromatin interaction as a switch for DIM2-mediated DNA methylation
Source: Nat Commun. 2024 Aug 9;15:6815. doi: 10.1038/s41467-024-51246-4 (PMC11315935; doi:10.1038/s41467-024-51246-4)
Supplement: Supplementary file 1 — Supplementary Information [file 41467_2024_51246_MOESM1_ESM.pdf]

Supplementary Information for

Multi-layered heterochromatin interaction as a switch for DIM2-mediated DNA methylation

Zengyu Shao[1], Jiuwei Lu[1], Nelli Khudaverdyan[1], Jikui Song[1,#]

[1]Department of Biochemistry, University of California, Riverside, CA 92521, USA

[#]Correspondence: jikui.song@ucr.edu

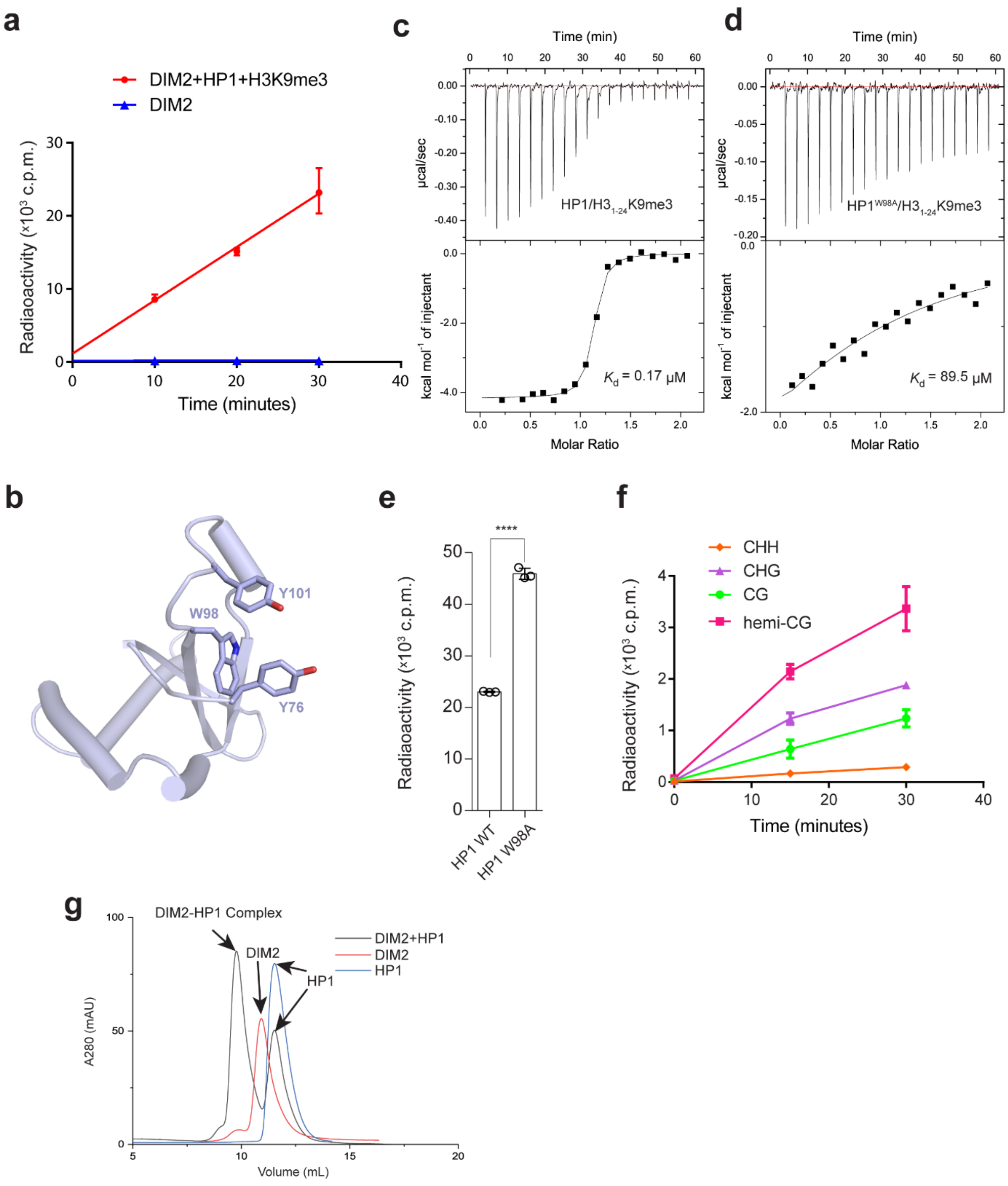

Supplementary Fig. 1 | Enzymatic and biochemical analysis of DIM2.

(**a**) In vitro DNA methylation assay of DIM2 on a $(CTA)_{12}/(TAG)_{12}$ DNA, in the presence or absence of HP1 and/or histone $H3_{1\text{-}24}K9me3$ peptide. Source data are provided as a Source Data file. (**b**) AlphaFold model of the HP1 chromodomain in *Neurospora Crassa*. The potential protein residues

forming H3K9me3-binding pocket are shown in stick representation. (**c,d**) Representative ITC binding curves for WT (c) or W98A-mutated (d) HP1 with the H3K9me3 peptide. The top panels show the raw thermogram data. The lower panel shows the integrated binding isotherms as a function of the molar ratio of the ligand to the enzyme. (**e**) In vitro DNA methylation assay of HP1, WT or W98A mutant, on a $(CTA)_{12}/(TAG)_{12}$ DNA duplex. Data are mean ± s.d. (n = 3 biological replicates). Statistical analysis used two-tailed Student's t test. ****$p < 0.0001$. Source data are provided as a Source Data file. (**f**) In vitro DNA methylation kinetics of DIM2 on DNA substrates containing one central CG, hemimethylated CG (hemi-CG), CHG or CHH target site. Source data are provided as a Source Data file. (**g**) Size-exclusion chromatography analysis of DIM2, HP1 and DIM2-HP1 complex.

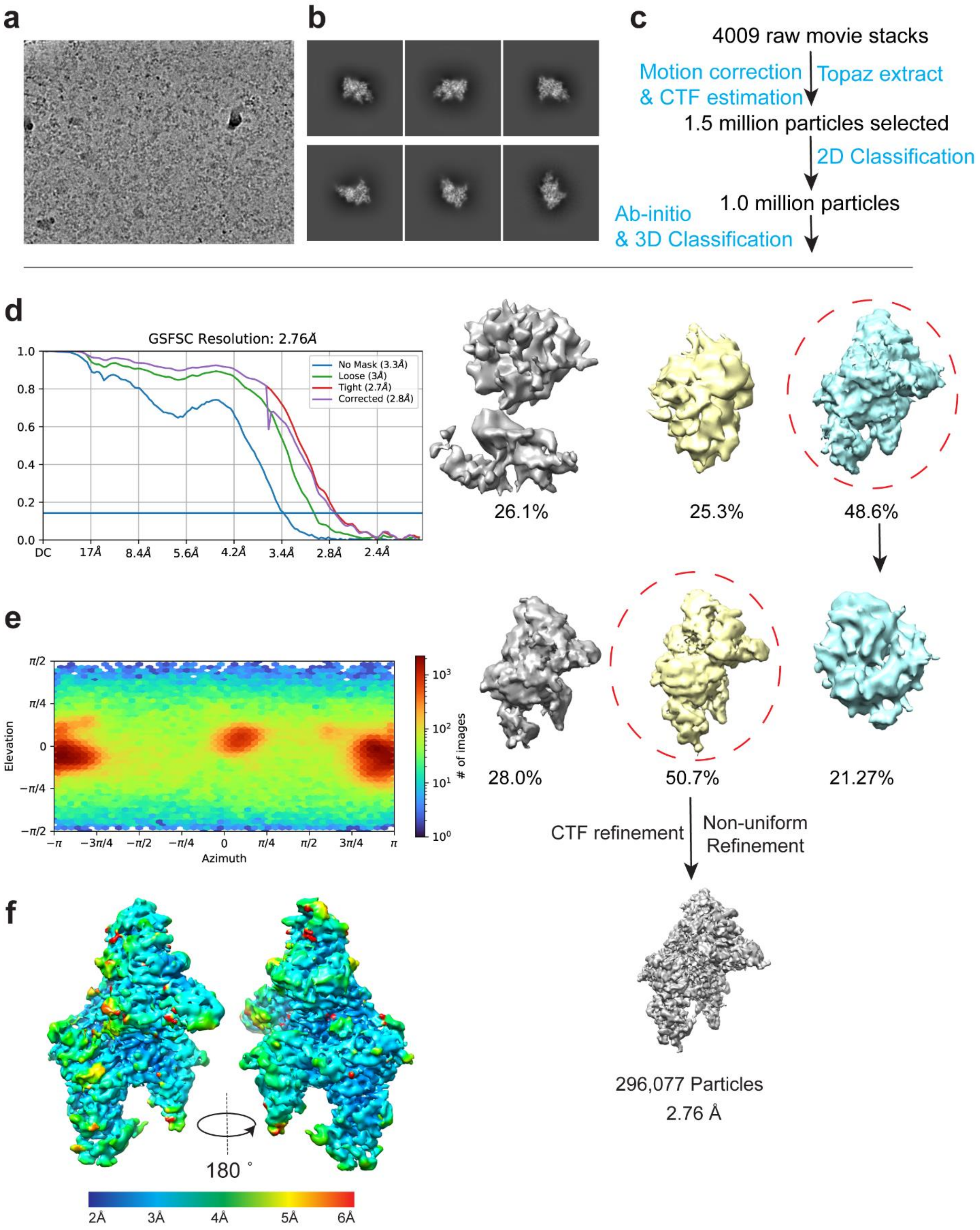
a
b
c
4009 raw movie stacks
Motion correction
& CTF estimation
Topaz extract
1.5 million particles selected
2D Classification
1.0 million particles
Ab-initio
& 3D Classification
d
GSFSC Resolution: 2.76Å
No Mask (3.3Å)
Loose (3Å)
Tight (2.7Å)
Corrected (2.8Å)
26.1%
25.3%
48.6%
e
Elevation
Azimuth
# of images
28.0%
50.7%
21.27%
CTF refinement
Non-uniform
Refinement
f
180 °
2Å
3Å
4Å
5Å
6Å
296,077 Particles
2.76 Å

Supplementary Fig. 2 | Data processing workflow for cryo-EM reconstruction of DIM2-HP1 complex.

(**a**) A representative micrograph of the DIM2-HP1complex by cryo-EM. (**b**) Representative 2D classes of the DIM2-HP1 complex from CryoSPARC. (**c**) A simplified flow chart of cryo-EM data processing. (**d**) Fourier shell correlation (FSC) curve of DIM2-HP1complex map as a function of resolution using CryoSPARC output. (**e**) Angular distribution calculated in CryoSPARC for particle projections. Heat map shows number of particles for each viewing angle. (**f**) Local resolution map of the DIM2-HP1 complex.

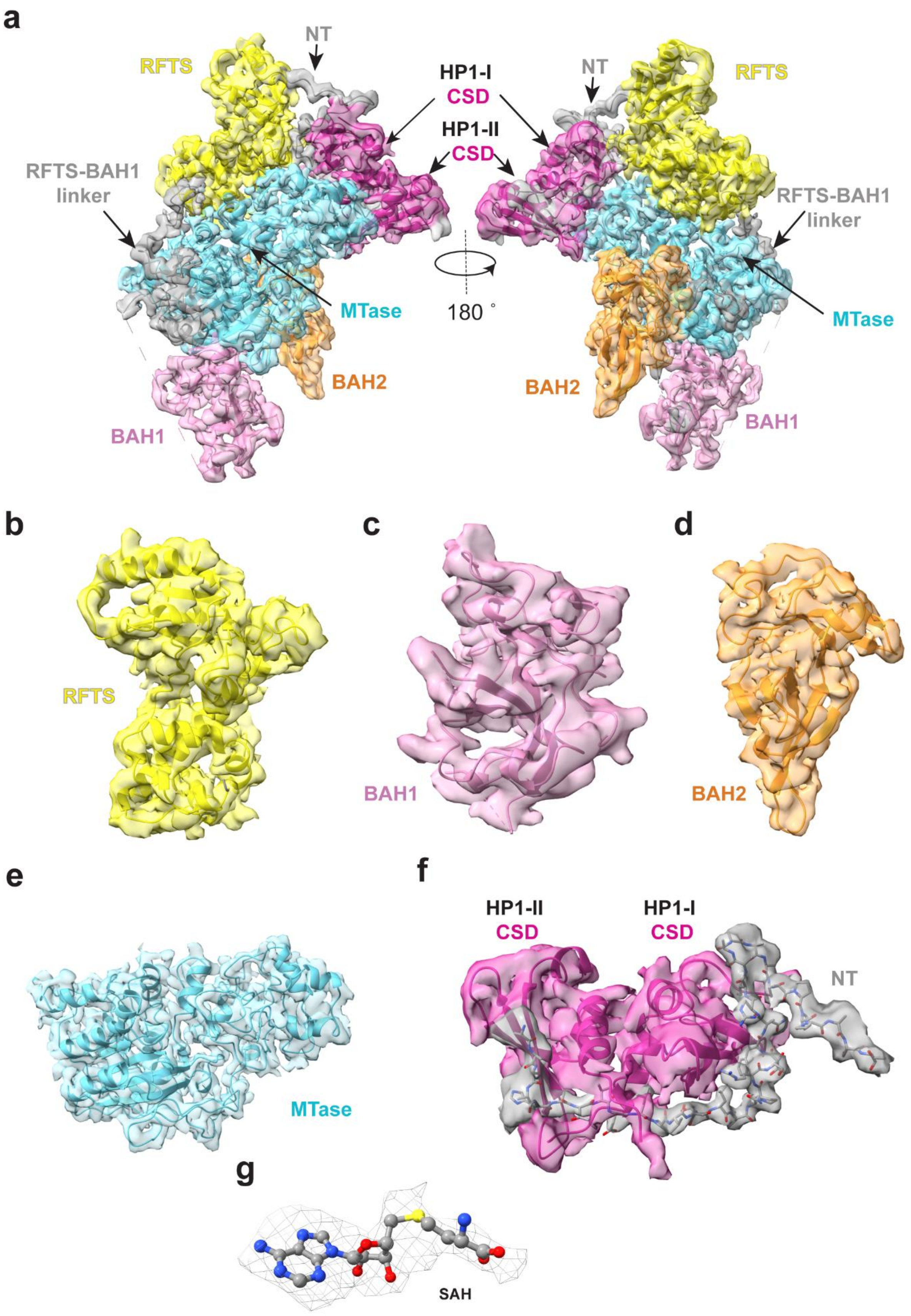
a
NT
RFTS
HP1-I
CSD
HP1-II
CSD
NT
RFTS
RFTS-BAH1
linker
RFTS-BAH1
linker
MTase
180 °
MTase
BAH2
BAH2
BAH1
BAH1
b
c
d
RFTS
BAH1
BAH2
e
f
HP1-II
CSD
HP1-I
CSD
NT
MTase
g
SAH

Supplementary Fig. 3 | Cryo-EM density maps for the DIM2-HP1 complex.

(**a-g**) Cryo-EM density and built in models for the final map for the DIM2-HP1 complex (a), DIM2 RFTS domain (b), DIM2 BAH1 domain (c), DIM2 BAH2 domain (d), DIM2 MTase domain (e), HP1 dimer and bound DIM2 N-terminal tail (f), and SAH molecule in a different view from that of Fig. 1g (g).

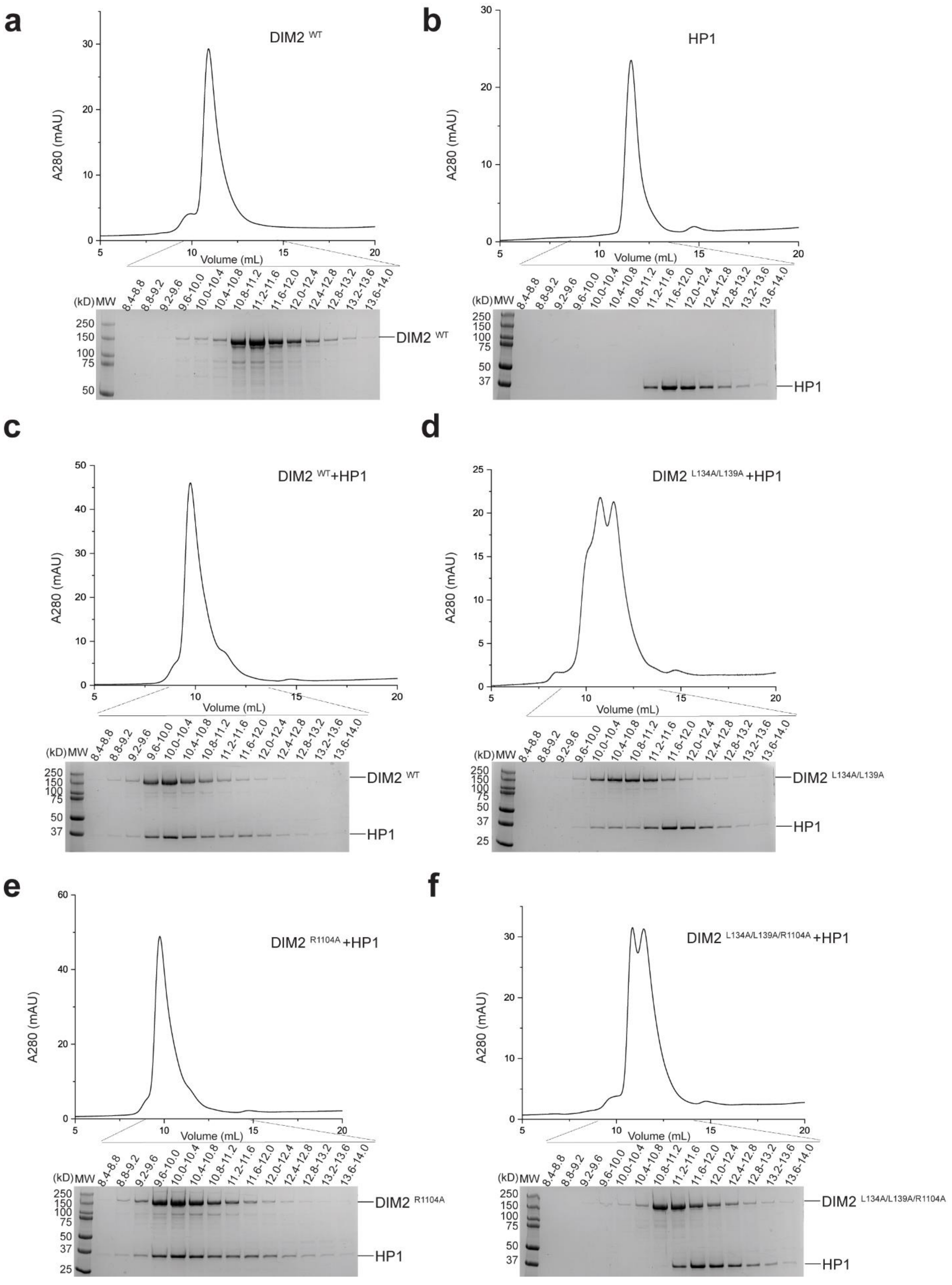
a
DIM2 WT
A280 (mAU)
Volume (mL)
(kD) MW
8.4-8.8
8.8-9.2
9.2-9.6
9.6-10.0
10.0-10.4
10.4-10.8
10.8-11.2
11.2-11.6
11.6-12.0
12.0-12.4
12.4-12.8
12.8-13.2
13.2-13.6
13.6-14.0
250
150
100
75
50
DIM2 WT
b
HP1
A280 (mAU)
Volume (mL)
HP1
c
DIM2 WT+HP1
A280 (mAU)
Volume (mL)
DIM2 WT
HP1
d
DIM2 L134A/L139A +HP1
A280 (mAU)
Volume (mL)
DIM2 L134A/L139A
HP1
e
DIM2 R1104A +HP1
A280 (mAU)
Volume (mL)
DIM2 R1104A
HP1
f
DIM2 L134A/L139A/R1104A +HP1
A280 (mAU)
Volume (mL)
DIM2 L134A/L139A/R1104A
HP1

Supplementary Fig. 4 | Size-exclusion chromatography analyses of the DIM2-HP1 interaction.

(**a,b**) Size-exclusion chromatography profile of WT DIM2 ($DIM2^{WT}$) alone (a) or HP1 alone (b). (**c-f**) Size-exclusion chromatograph profile of the DIM2-HP1 mixture harboring $DIM2^{WT}$ (c), L134A/L139A-mutated DIM2 ($DIM2^{L134A/L139A}$) (d), R1104A-mutated DIM2 ($DIM2^{R1104A}$) (e), and L134A/L139A/R1104A-mutated DIM2 ($DIM2^{L134A/L139A/R1104A}$) (f). The SDS-PAGE images for selected fractions are shown below.

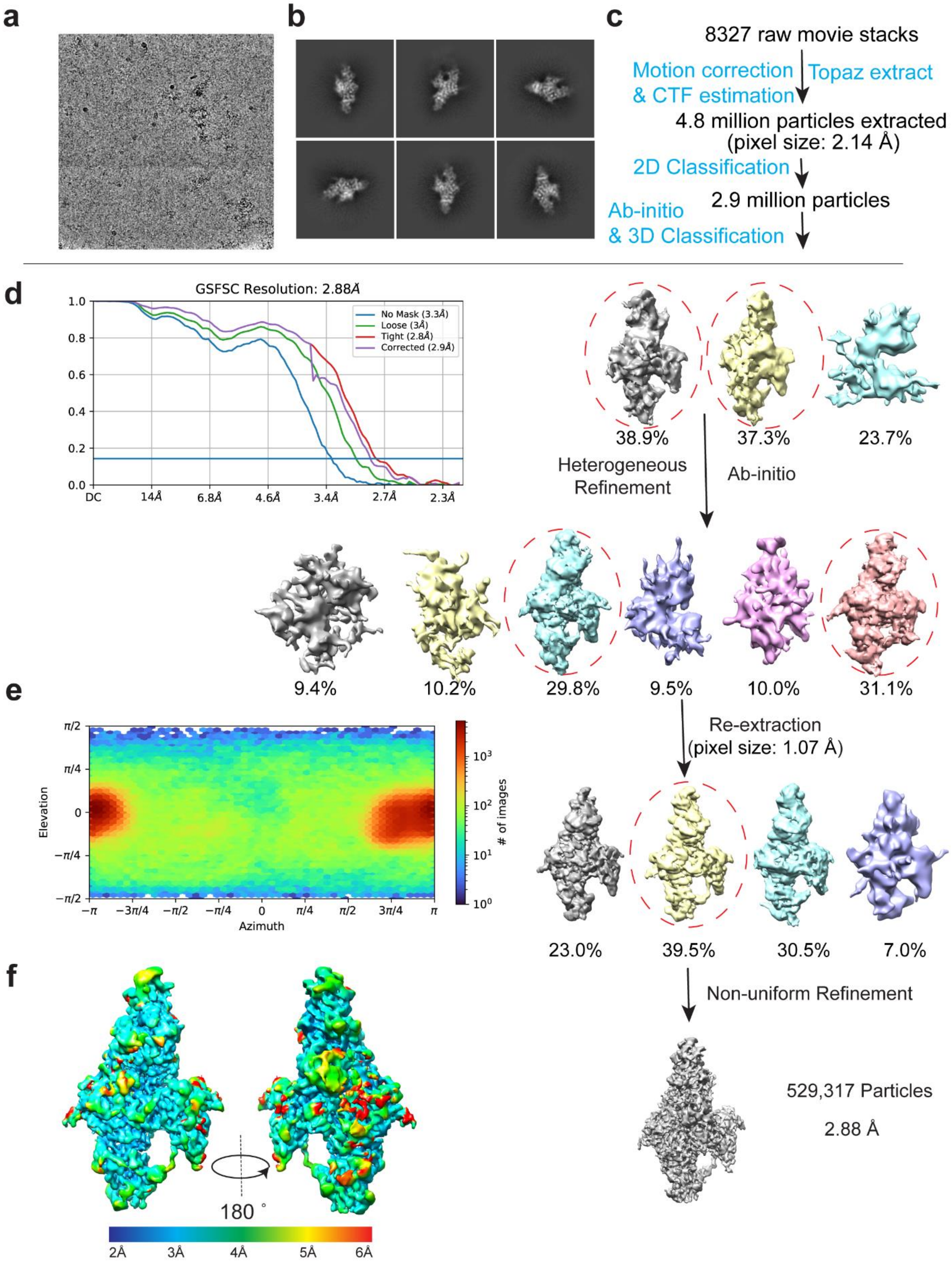

Supplementary Fig. 5 | Data processing workflow for cryo-EM reconstruction of apo-DIM2.

(**a**) A representative micrograph of apo-DIM2 by cryo-EM. (**b**) Representative 2D classes of apo-DIM2 from CryoSPARC. (**c**) A simplified flow chart of cryo-EM data processing. (**d**) Fourier shell correlation (FSC) curve of apo-DIM2 map as a function of resolution using CryoSPARC output. (**e**) Angular distribution calculated in CryoSPARC for particle projections. Heat map shows number of particles for each viewing angle. (**f**) Local resolution map of apo-DIM2.

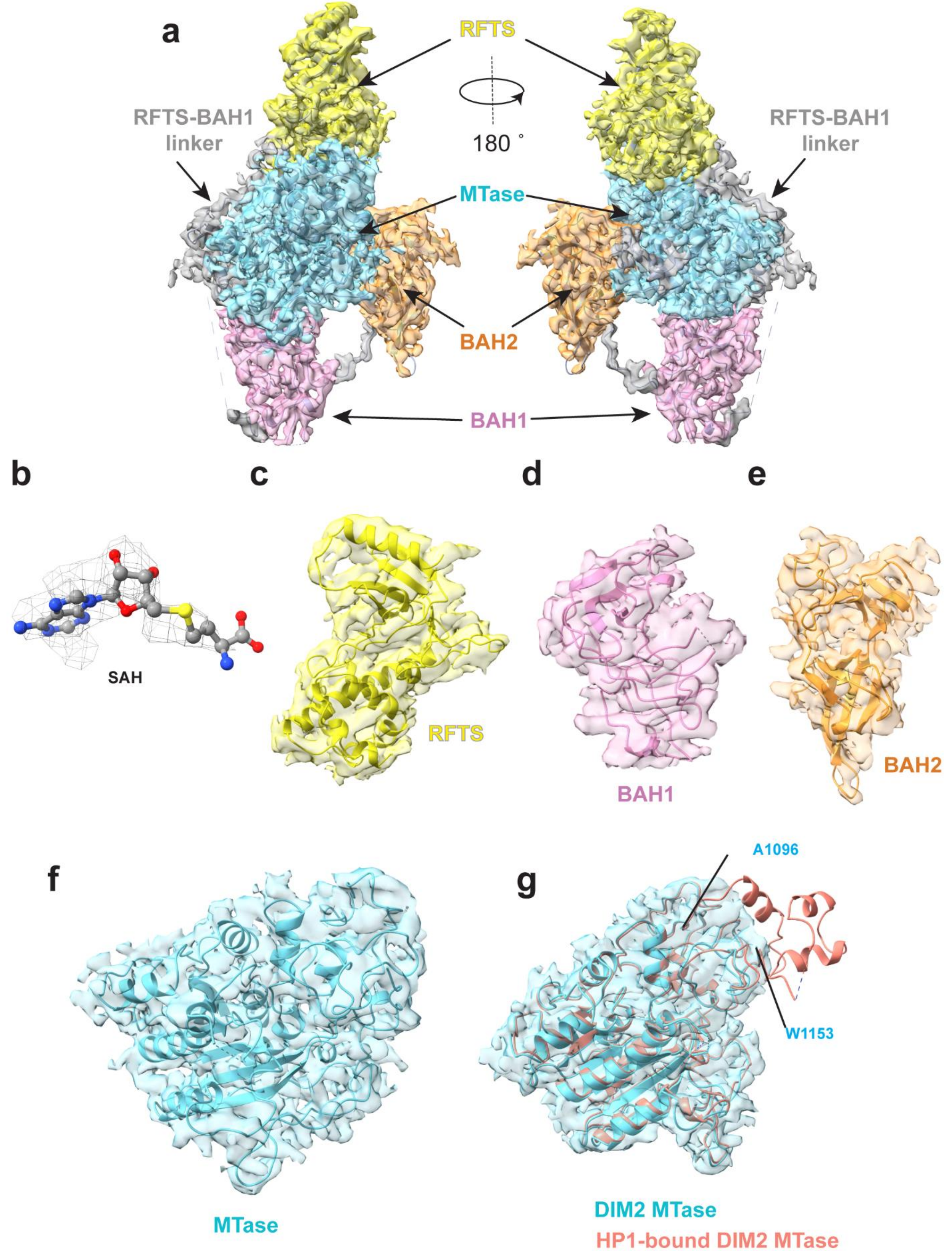
a
RFTS
180 °
RFTS-BAH1 linker
RFTS-BAH1 linker
MTase
BAH2
BAH1
b
SAH
c
RFTS
d
BAH1
e
BAH2
f
MTase
g
A1096
W1153
DIM2 MTase
HP1-bound DIM2 MTase

Supplementary Fig. 6 | Cryo-EM density maps for apo-DIM2.

(**a-f**) Cryo-EM density and built in models for the final map for apo-DIM2 (a), SAH molecule in a different view from Fig. 3e (b), DIM2 RFTS domain (c), DIM2 BAH1 domain (d), DIM2 BAH2 domain (e), and DIM2 MTase domain (f). (**g**) Structural alignment between HP1-bound DIM2 MTase (salmon) and the MTase domain of apo-DIM2 (cyan).

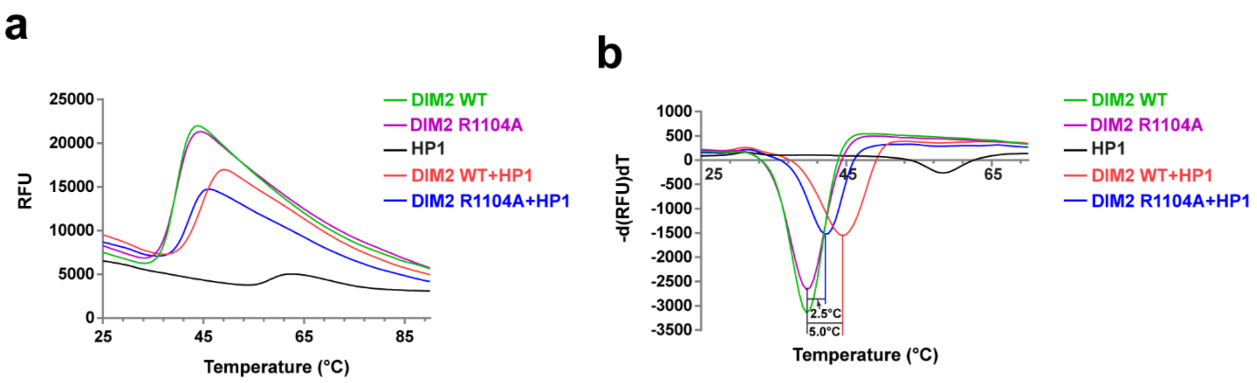

Supplementary Fig. 7 | Thermal shift assay for wild type (WT) and R1104A-mutated DIM2, in the presence or absence of HP1.

(**a,b**) Raw fluorescence data (a) and first derivative of the raw data (b). The difference in melting temperature (Tm) between free and complexed WT or R1104A DIM2 is indicated, respectively. Note that HP1 alone (black) in two-fold molar excess than that of DIM2 (green and purple) yielded a much-reduced fluorescence signal, with a Tm of 58.5 °C, which is distinct from the major fluorescence peaks measured for the free or HP1-bound state of DIM2.

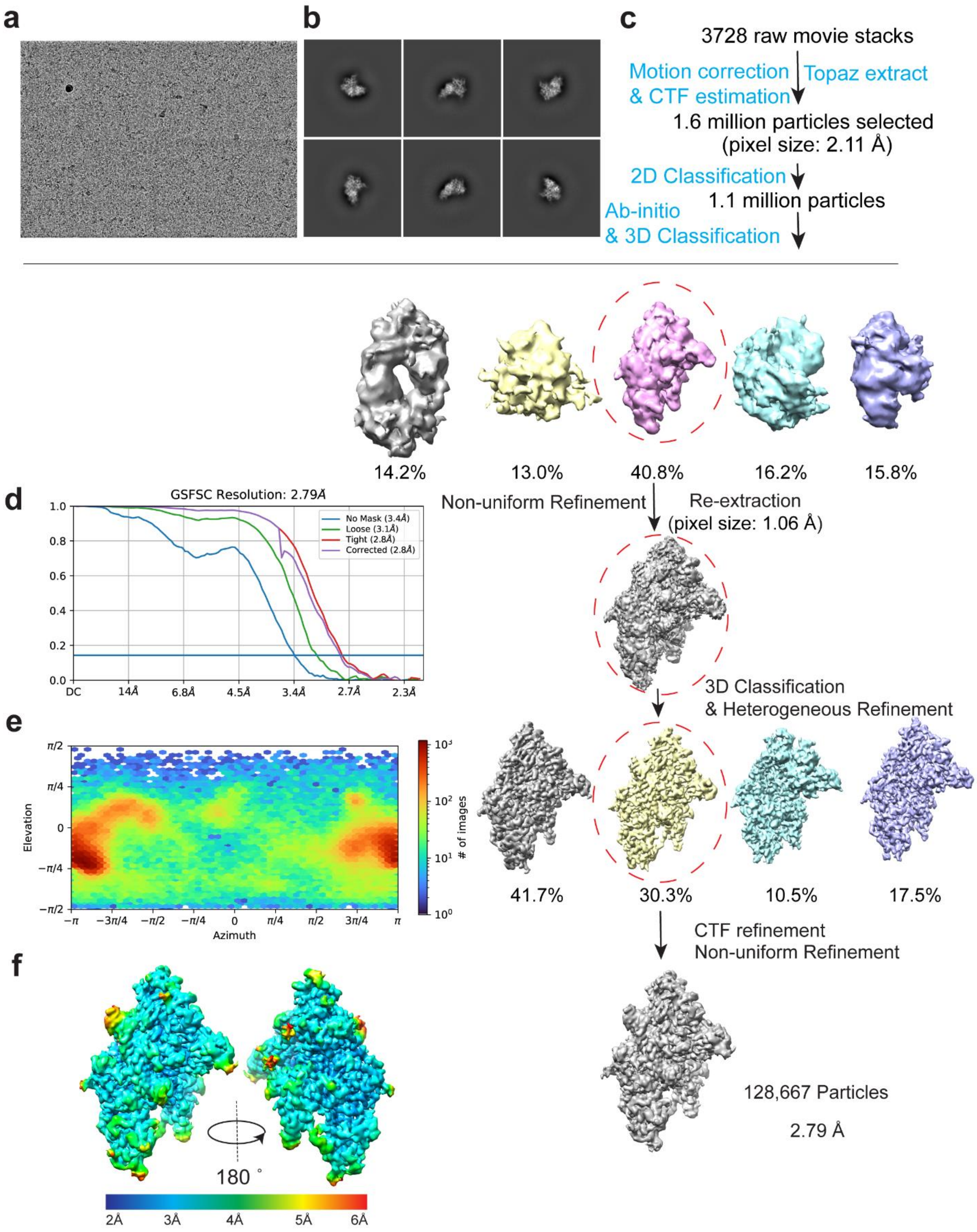
a
b
c
3728 raw movie stacks
Motion correction
& CTF estimation
Topaz extract
1.6 million particles selected
(pixel size: 2.11 Å)
2D Classification
1.1 million particles
Ab-initio
& 3D Classification
14.2%
13.0%
40.8%
16.2%
15.8%
Non-uniform Refinement
Re-extraction
(pixel size: 1.06 Å)
3D Classification
& Heterogeneous Refinement
41.7%
30.3%
10.5%
17.5%
CTF refinement
Non-uniform Refinement
128,667 Particles
2.79 Å
d
GSFSC Resolution: 2.79Å
No Mask (3.4Å)
Loose (3.1Å)
Tight (2.8Å)
Corrected (2.8Å)
DC
14Å
6.8Å
4.5Å
3.4Å
2.7Å
2.3Å
e
Elevation
Azimuth
# of images
f
180 °
2Å
3Å
4Å
5Å
6Å

Supplementary Fig. 8 | Data processing workflow for cryo-EM reconstruction of DIM2-HP1-H3K9me3-DNA complex.

(**a**) A representative micrograph of the DIM2-HP1-H3K9me3-DNA by cryo-EM. (**b**) Representative 2D classes of the DIM2-HP1-H3K9me3-DNA from CryoSPARC. (**c**) A simplified flow chart of cryo-EM data processing. (**d**) Fourier shell correlation (FSC) curve of the DIM2-HP1-H3K9me3-DNA map as a function of resolution using CryoSPARC output. (**e**) Angular distribution calculated in CryoSPARC for particle projections. Heat map shows number of particles for each viewing angle. (**f**) Local resolution map of DIM2-HP1-H3K9me3-DNA complex.

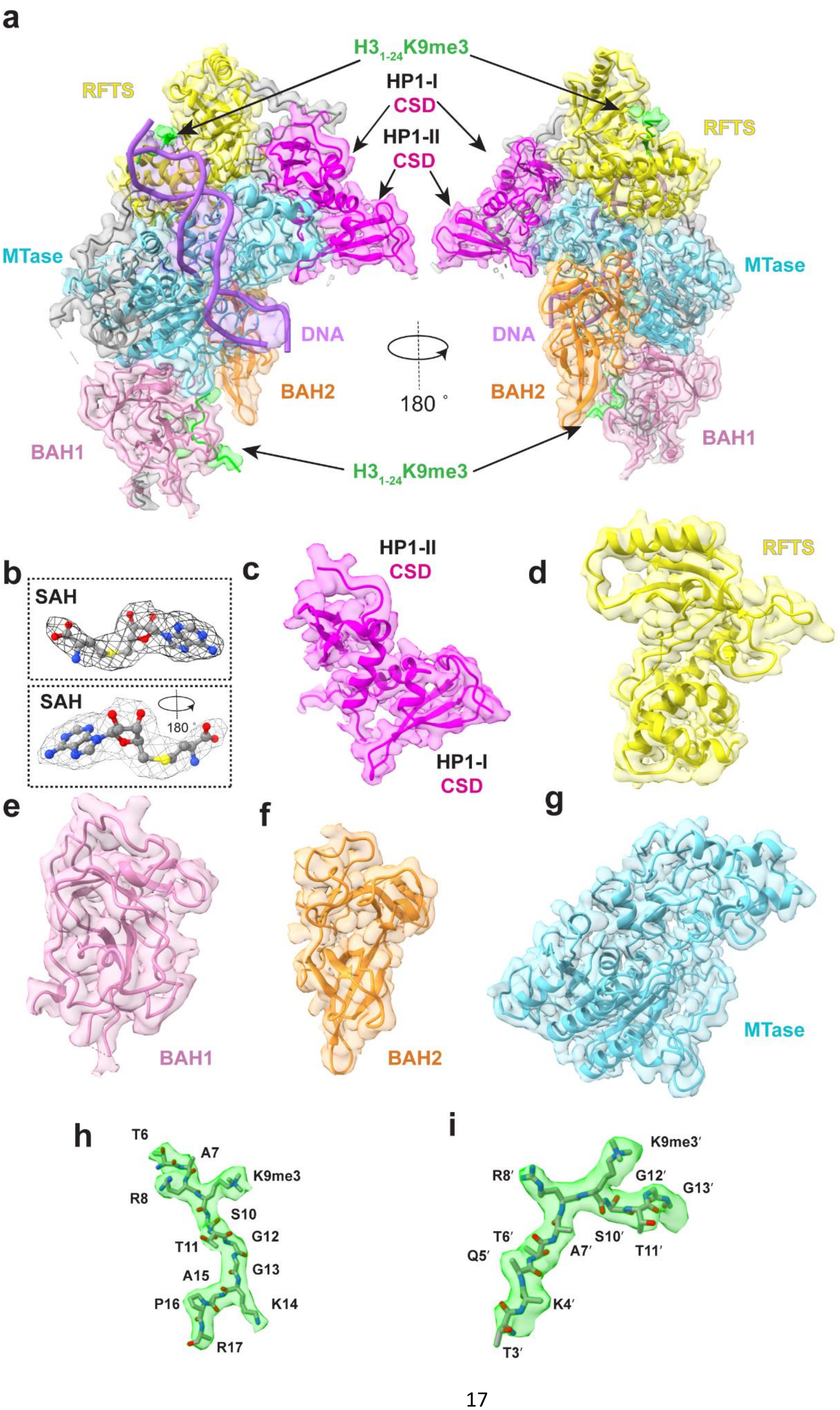
a
H3$_{1-24}$K9me3
HP1-I
CSD
HP1-II
CSD
RFTS
MTase
DNA
BAH2
BAH1
180 °
b
SAH
SAH
180 °
c
HP1-II
CSD
HP1-I
CSD
d
RFTS
e
BAH1
f
BAH2
g
MTase
h
T6
A7
K9me3
R8
S10
G12
T11
A15
G13
P16
K14
R17
i
K9me3′
R8′
G12′
G13′
T6′
S10′
Q5′
A7′
T11′
K4′
T3′

Supplementary Fig. 9 | Cryo-EM density maps for the DIM2-HP1-H3K9me3-DNA complex.

(**a-i**) Cryo-EM density and built in models for the final map for the DIM2-HP1-H3K9me3-DNA complex (a), SAH molecule in two opposite views (b), HP1 dimer (c), DIM2 RFTS domain (d), DIM2 BAH1 domain (e), DIM2 BAH2 domain (f), DIM2 MTase domain (g), DIM2 RFTS-bound H3K9me3 (h), and DIM2 BAH1-bound H3K9me3 (i).

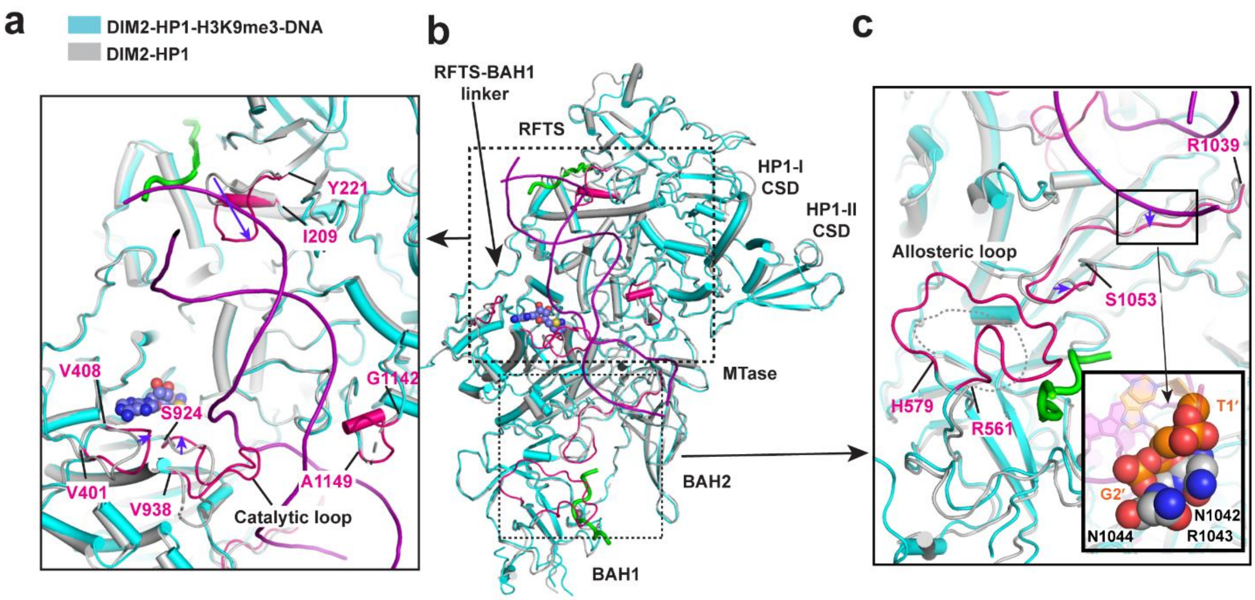

Supplementary Fig. 10 | Structural comparison of DIM2-HP1-H3K9me3-DNA complex and DIM2-HP1 complex.

(**a-c**) Structural overlay of the DIM2-HP1 complex (grey) and the DIM2-HP1-H3K9me3-DNA complex (cyan). The DIM2 regions that exhibit the largest conformational changes are colored in hot pink in the DIM2-HP1-H3K9me3-DNA complex and highlighted in expanded views (a) and (c). The potential steric clashes between the bridging loop of DIM2 and DNA are indicated in sphere representation in (c).

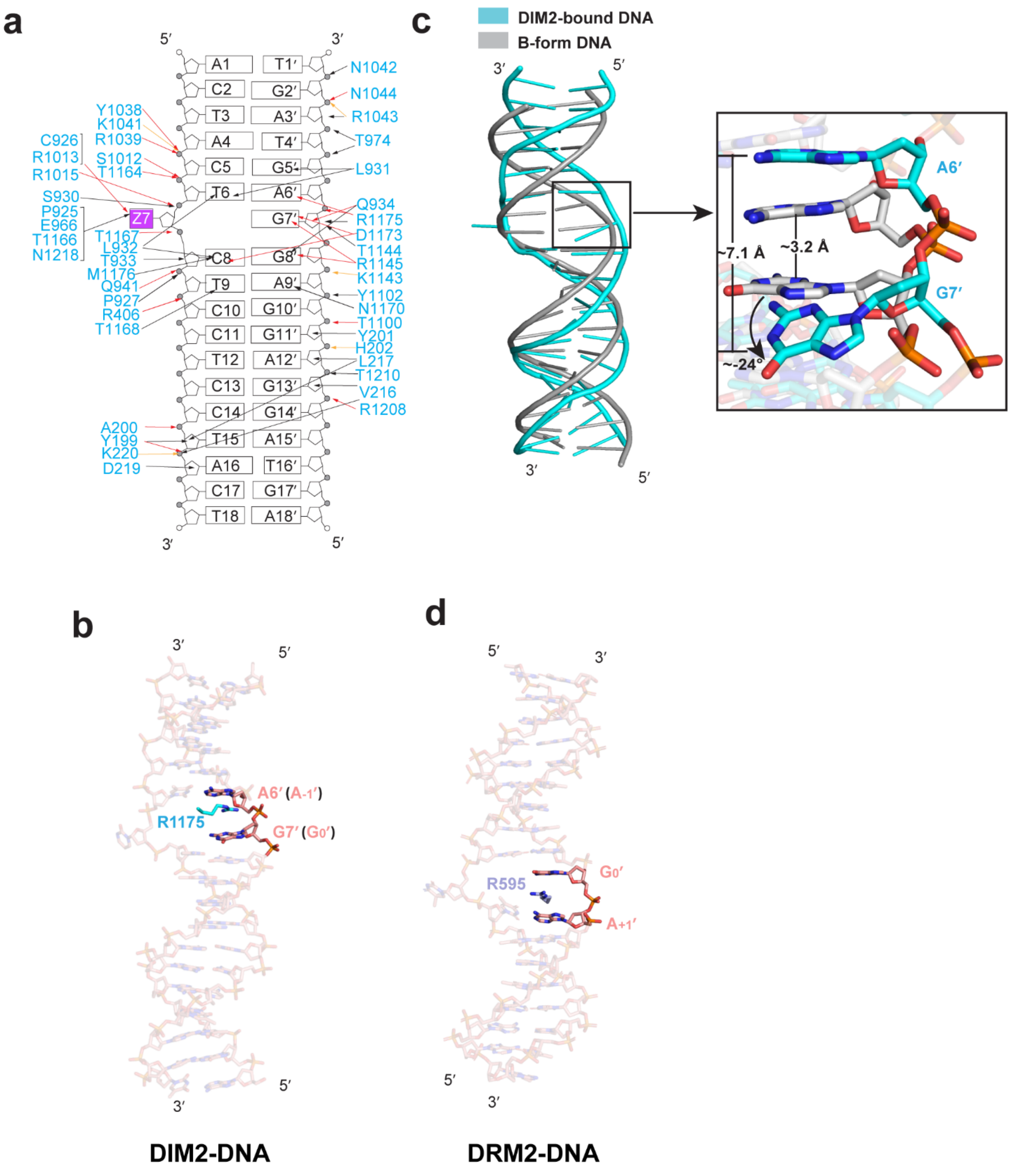

Supplementary Fig. 11 | Structural details for the DIM2-DNA interaction.

(**a**) Schematic view of the DIM2-DNA interactions. Hydrogen-bonding, electrostatic, and van der

Waals contacts are indicated by red, wheat, and black arrows, respectively. (**b**) Structural overview of the DNA intercalation by DIM2 R1175. Note that DIM2 R1175 intercalates between the orphan guanine G7′ (a.k.a. G_0') and its preceding nucleotide A6′ (a.k.a. A_{-1}'). (**c**) Structural alignment between DIM2-bound DNA (cyan) and B-form DNA (grey), with the expanded view showing that DIM2 binding increased the rise of the A6′-G7′ step from 3.2 Å in B-form DN A to 7.1 Å in DIM2-bound DNA and introduced a roll of ~-24°. (**d**) Structural overview of the structural intercalation by DRM2 R595 (PDB 7L4C). Note that DRM2 R595 intercalates between the orphan guanine G_0' and its subsequent nucleotide A_{+1}'.

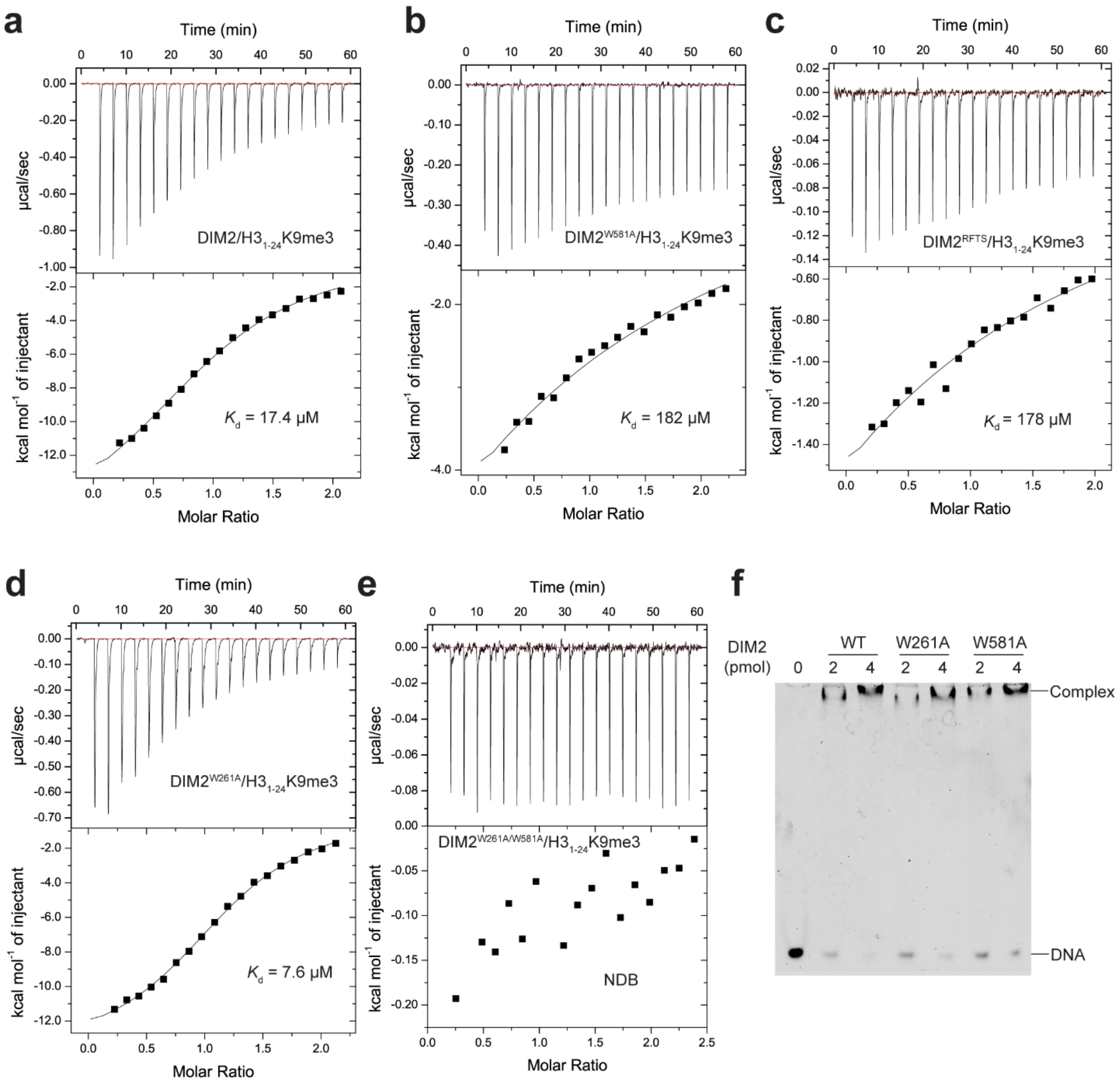

Supplementary Fig. 12 | Biochemical analysis of DIM2, WT or mutant.

(**a-e**) Representative ITC binding curves for the H3K9me3 peptides to the DIM2 (a), W581A-mutated DIM2 ($DIM2^{W581A}$) (b), DIM2 RFTS domain ($DIM2^{RFTS}$) (c), W261A-mutated DIM2 ($DIM2^{W261A}$) (d), and W261A/W581A-mutated DIM2 ($DIM2^{W261A/W581A}$) (e). The top panels show the raw heat change data. The lower panel shows the integrated binding isotherms as a function

of the molar ratio of the ligand to the enzyme. (**f**) EMSA analysis of the interaction between DIM2 and an 18-bp DNA in the presence of the H3K9me3 peptide. Each sample contains 1 pmol of DNA, 0 (for the sample lack of DIM2) or 8 (for the sample containing DIM2) pmol of H3K9me3 peptide, and various amounts of DIM2 protein. The bands corresponding to free DNA and the protein-DNA complex are indicated, respectively. The experiment was repeated three times with consistent results. Source data are provided as a Source Data file.

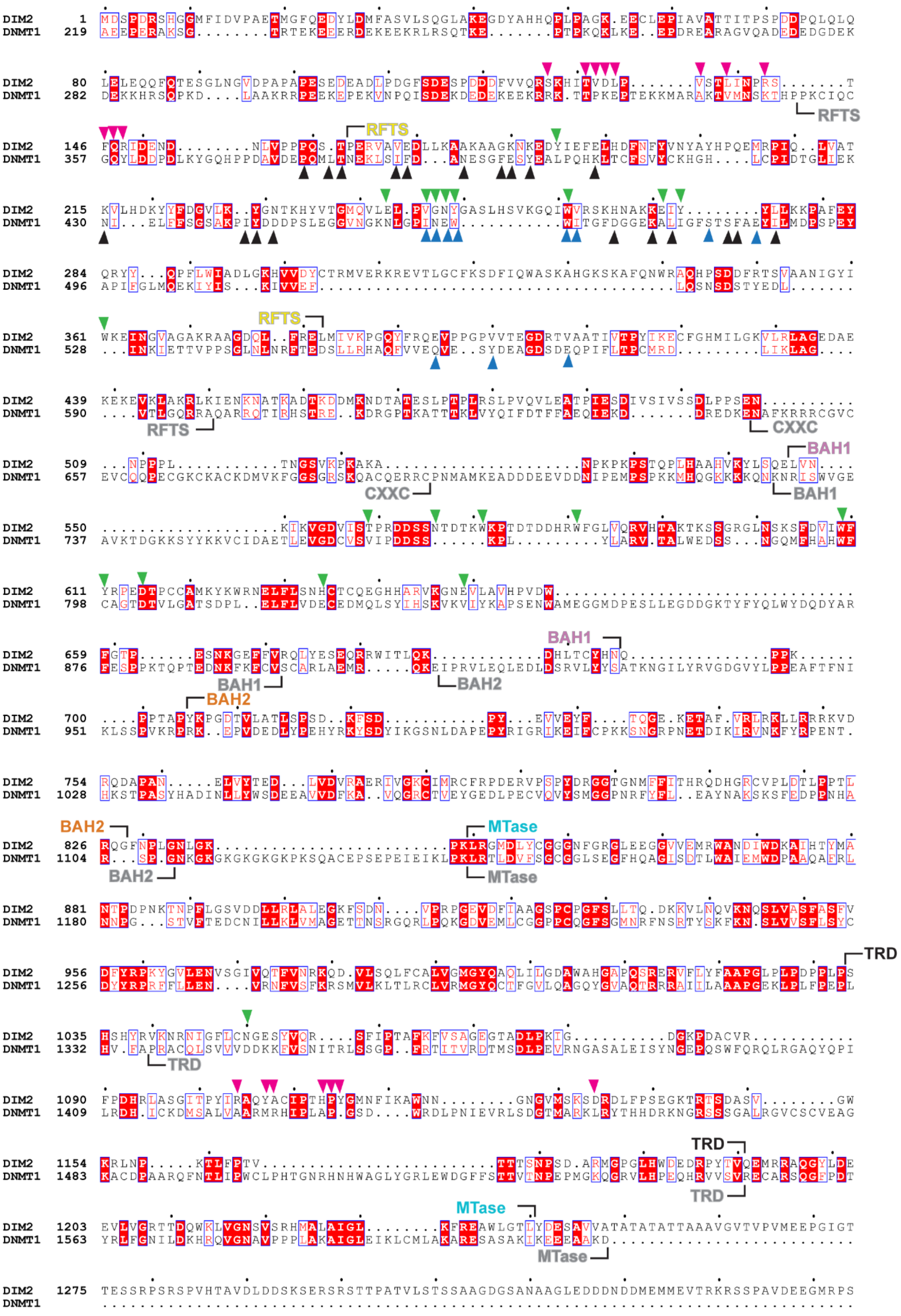
DIM2
DNMT1
RFTS
CXXC
BAH1
BAH2
MTase
TRD

Supplementary Fig. 13 | Sequence alignment between DIM2 and human DNMT1.

Identical or similar residues are boxed and colored in red. Completely conserved residues are shaded in red. The DIM2 domains are labeled above the corresponding sequences, while the DNMT1 domains are labeled below the corresponding sequences. The DIM2 residues responsible for the H3K9me3 and HP1 interactions are marked by green and magenta arrows, respectively. The DNMT1 residues responsible for the interaction with H3K9me3 and ubiquitin are marked by blue and dark arrows, respectively.

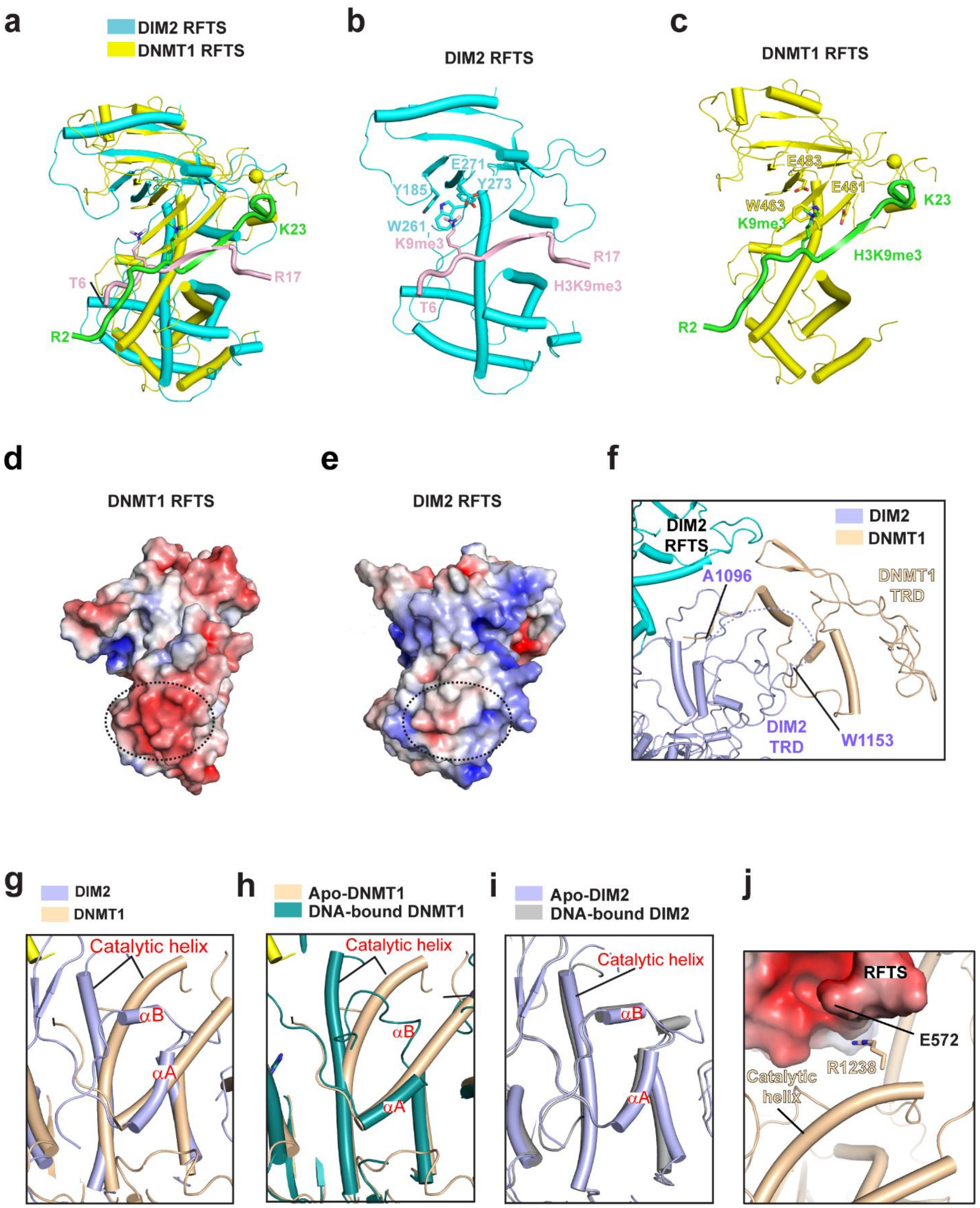

Supplementary Fig. 14 | Structural comparison between the RFTS domains of DIM2 and DNMT1.

(**a**) Structural alignment of H3K9me3 (light pink)-bound DIM2 RFTS domain (cyan) and the H3K9me3 (green)-bound DNMT1 RFTS domain (yellow) (PDB 6PZV). The zinc ion bound to the DNMT1 RFTS domain is shown in sphere representation. (**b**) Ribbon representation of H3K9me3 (light pink)-bound DIM2 RFTS domain (cyan). The H3K9me3 pocket residues of DIM2 RFTS are shown in stick representation. (**c**) Ribbon representation of H3K9me3 (green)-bound DNMT1 RFTS domain (yellow) (PDB 6PZV). The H3K9me3 pocket residues are shown in stick representation. (**d**) Electrostatic surface of the DNMT1 RFTS domain (PDB 4WXX). The region involved in the binding to the MTase domain is circled by dotted line. (**e**) Electrostatic surface of the DIM2 RFTS domain. The region corresponding to the MTase domain-binding site of the DNMT1 RFTS domain is circled by dotted line. (**f**) Close-up view of the overlaid TRD subdomain between apo-form DIM2 and DNMT1 (PDB 4WXX). (**g**) Structural overlay between the catalytic helix and RFTS-BAH1 linker helices αA and αB of apo-DIM2 and the corresponding regions of DNA-free DNMT1 (PDB 4WXX). (**h**) Structural overlay between apo-form (PDB 4WXX) and DNA-bound (PDB 7XI9) DNMT1, highlight the region corresponding to the catalytic helix and RFTS-BAH1 linker helices αA and αB of DIM2. (**i**) Structural overlay between apo-DIM2 and DNA-bound DIM2, highlight the catalytic helix and RFTS-BAH1 linker helices αA and αB. (**j**) Electrostatic contact between residue E572 in the RFTS domain (Electrostatic surface) and residue R1238 (stick representation) in the catalytic helix of DNMT1 (ribbon representation).

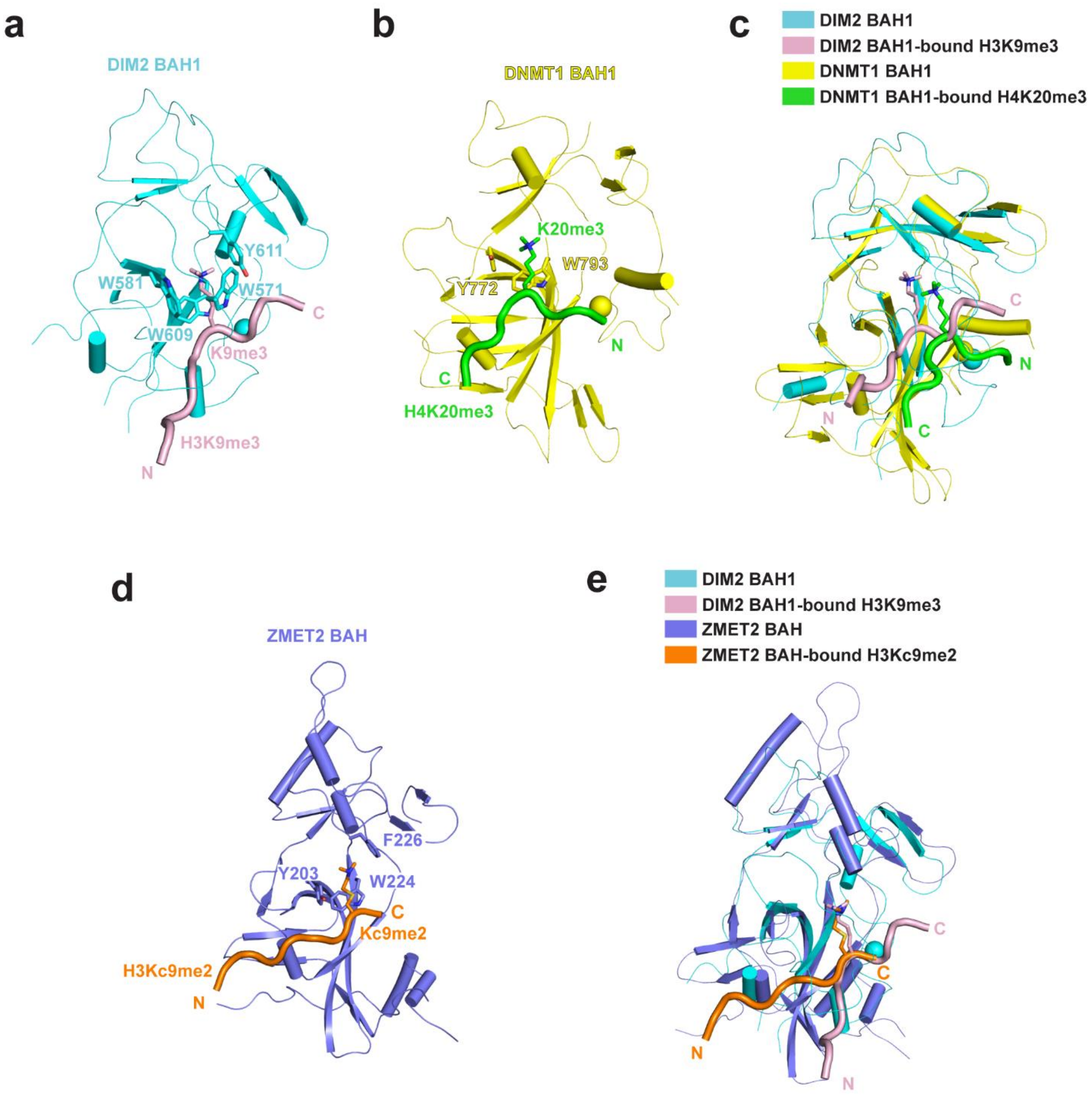

Supplementary Fig. 15 | Structural comparison of the histone-binding BAH domains between DIM2, DNMT1, and ZMET2.

(**a**) Ribbon representation of H3K9me3 (light pink)-bound DIM2 BAH1 domain (cyan), with the residues forming the H3K9me3-binding cage shown in stick representation. The zinc ion is shown in sphere representation. The N- and C-termini of the peptide are labeled "N" and "C", respectively. (**b**) Ribbon representation of H4K20me3 (green)-bound DNMT1 BAH1 domain (yellow) (PDB

7LMK), with the residues forming the H4K20me3-binding cage shown in stick representation. The zinc ion is shown in sphere representation. The N- and C-termini of the peptide are labeled “N” and “C”, respectively. (**c**) Structural overlay of H3K9me3-bound BAH1 (cyan) of DIM2 with H4K20me3-bound DNMT1 BAH1 (yellow). The H3K9me3 and H4K20me3 are shown in pink and green, respectively. (**d**) Ribbon representation of H3K9me3 (orange) -bound ZMET2 BAH domain (slate) (PDB 7UBU), with the residues forming the H3K9me2-binding cage shown in stick representation. (**e**) Structural overlay of H3K9me3 (pink)-bound BAH1 (cyan) of DIM2 with H3K9me2 (orange)-bound ZMET2 BAH domain (slate).

Supplementary Table 1. Summary of ITC binding parameters.

Protein	Peptide	K_d (μM)	N value
DIM2, WT	$H3_{1-24}K9me3$	17.4 ± 0.6#	0.95 ± 0.08
DIM2, W261A	$H3_{1-24}K9me3$	7.6 ± 1.2#	1.07 ± 0.11
DIM2, W581A	$H3_{1-24}K9me3$	182 ± 67#	1.0*
DIM2 RFTS domain	$H3_{1-24}K9me3$	178 ± 24**	1.0*
DIM2, W261A/W581A	$H3_{1-24}K9me3$	NDB	
HP1, WT	$H3_{1-24}K9me3$	0.17 ± 0.03#	0.97 ± 0.12
HP1, W98A	$H3_{1-24}K9me3$	89.5 ± 7.6#	1.03 ± 0.03

NDB, no detectable binding. * The N value was set manually. # The mean value and S.D. were derived from two-independent measurements. ** The mean value and S.D. were derived from curve fitting of a single measurement.

Supplementary Table 2. Cryo-EM data collection, refinement, and validation statistics.

States<br><br>Codes	**DIM2-HP1** (EMD-44415, PDB 9BAZ)	**Apo-DIM2** (EMD-44110, PDB 9BAP)	**DIM2-HP1-H3K9me3-DNA** (EMD-44111, PDB 9BAQ)
**Data collection and processing**			
Microscope	Titan Krios	Titan Krios	Titan Krios
Camera	K3 BioQuantum	K3 BioQuantum	K3 BioQuantum
Magnification	81,000	81,000	81,000
Voltage (kV)	300	300	300
Defocus range (μm)	-0.8 ~ -2.5	-0.6~ -1.5	-0.8 ~ -2.5
Exposure time (s)	4.1	2.4	2.7
Dose rate(e^{-}/Å^2/s)	16.3	20.8	25.9
Number of frames	68	40	30
Pixel size (Å)	1.056	1.07	1.056
Micrographs (no.)	4009	8327	3728
Symmetry imposed	*C1*	*C1*	*C1*
Initial particles (no.)	1,476,534	4,792,885	1,550,688
Final particles (no.)	296,077	529,317	128,667
Map resolution (Å)	2.76	2.88	2.79
FSC threhold	0.143	0.143	0.143
**Refinement**			
Initial model used	DIM2 (AlphaFold), HP1 (AlphaFold)	DIM2 (AlphaFold)	DIM2 (AlphaFold), HP1 (AlphaFold), DNA (PDB 6F57)
Model resolution (Å)	3.2	3.2	3.0
FSC threshold	0.5	0.5	0.5
Map sharpening *B* factor (Å^2)	-85.8	-107.3	n.a
CC (mask)	0.71	0.67	0.81
Model composition			
Non-hydrogen atoms	8283	6535	9733
Protein residues	1090	887	1154
DNA	-	-	36
Ion (zinc)	1	1	1
SAH	1	1	1
B factors (Å^2)			
Protein	128.17	98.02	127.86
DNA	-	-	196.65
Ion (zinc)	188.74	153.67	196.94
SAH	114.25	85.7	111.6
R.m.s. deviations			
Bond lengths (Å)	0.004	0.003	0.003
Bond angles (°)	0.690	0.658	0.619
**Validation**			
MolProbity score	1.79	1.71	1.62
Clashscore	12.04	9.48	8.74
Poor rotamers (%)	0.5	0.84	0.66
Ramachandran plot			
Favored (%)	96.83	96.68	97.18
Allowed (%)	3.17	3.32	2.82
Disallowed (%)	0.00	0.00	0.00

n.a: Anisotropic sharpened by Phenix
